# Severe maternal morbidity and its associated factors: A cross-sectional study in Morang district, Nepal

**DOI:** 10.1371/journal.pone.0261033

**Published:** 2021-12-31

**Authors:** Sushma Rajbanshi, Mohd Noor Norhayati, Nik Hussain Nik Hazlina

**Affiliations:** 1 School of Medical Sciences, Women’s Health Development Unit, Universiti Sains Malaysia, Kubang Kerian, Malaysia; 2 Department of Family Medicine, School of Medical Sciences, Universiti Sains Malaysia, Kubang Kerian, Malaysia; Anglia Ruskin University, UNITED KINGDOM

## Abstract

**Background:**

Understanding maternal morbidity and its determinants can help identify opportunities to prevent obstetric complications and improvements for maternal health. This study was conducted to determine the prevalence of severe maternal morbidity (SMM) and the associated factors.

**Methods:**

A hospital-based cross-sectional study was conducted at Koshi Hospital, Nepal, from January to March 2020. All women who met the inclusion criteria of age ≥18 years of age, Morang residents of Nepalese nationality, had received routine antenatal care, and given birth at Koshi Hospital were recruited consecutively. The World Health Organization criteria were used to identify the women with SMM. A multiple logistic regression analysis was performed. Overall, 346 women were recruited.

**Findings:**

The prevalence of SMM was 6.6%. Among the SMM cases, the most frequently occurring SMM conditions were hypertensive disorders (12, 56.5%), hemorrhagic disorders (6, 26.1%), and severe management indicators (8, 34.8%). Women with no or primary education (adjusted odds ratio: 0.10, 95% confidence interval: 0.01, 0.76) decreased the odds of SMM compared to secondary education.

**Conclusion:**

The approximately 7% prevalence of SMM correlated with global studies. Maternal education was significantly associated with SMM. If referral hospitals were aware of the expected prevalence of potentially life-threatening maternal conditions, they could plan to avert future reproductive complications.

## Introduction

Maternal mortality is a public health problem studied worldwide [1], but the existing research on maternal mortality represents only a fraction of the problem [2]. Globally, the maternal mortality ratio has declined by 38% between 2000 and 2017; the greatest decrease during this period was in Southern Asia, with a nearly 60% reduction in maternal mortality ratio [3]. Maternal near-miss (MNM) and severe maternal morbidity (SMM) are new strategic indicators of maternal health conditions [4]. The World Health Organization (WHO) adopted and defined MNM and SMM standard criteria in 2009 [5]. The purpose of developing these uniform criteria was to provide common ground for comparisons across countries [5, 6]. MNM refers to “a woman who nearly died but survived a complication that occurred during pregnancy, childbirth or within 42 days of termination of pregnancy” [5]. The WHO working group has recommended the use of the term MNM as it best reflects the severity of events. However “severe acute maternal morbidity” (SAMM) is also used for MNM [5]. The WHO uses clinical-, laboratory-, and management-based criteria to identify MNM [7].

If observed across a broad spectrum, women’s reproductive health starts from a healthy pregnancy, morbidity, severe morbidity, near miss, and ends at maternal death. SMM lies somewhere between these two spectra before near-miss [8, 9]. Severe maternal morbidities are less in severity than MNM [5]. SMM includes women who did not necessarily have a critical illness but suffered complications related to pregnancy, delivery, and puerperium [5]. The WHO defines SMM as “potentially life-threatening conditions during pregnancy, childbirth, or after the termination of pregnancy from which maternal near-miss cases would emerge” and is assessed based on the four standard conditions, which are (i) hemorrhagic disorders, (ii) hypertensive disorders, (iii) other systemic disorders, and (iv) severe management indicators [5]. The terms “maternal near miss” and “severe maternal morbidity” are used interchangeably in the literature, but SMM reflects a less severe condition than MNM [9, 10]. While both “SMM” and “potentially life-threatening conditions” are used, SMM will be applied in this study.

The extent of MNM has been studied widely [11]; however, limited studies are available on SMM. In Nepal, reported MNM prevalence ranged from 3.8 per 1000 live births in 2013 [12] to a maximum of 23.1 per 1000 deliveries in 2010 [13], while none are reported on SMM. Many studies, especially those in low-income countries, have used a modified version of the WHO near-miss approach, mainly due to its limited applicability in low-income settings, notably due to the laboratory- and management-based criteria [14, 15].

It is necessary to determine a relevant maternal morbidity measurement and investigate its associated factors to improve maternal healthcare services because maternal deaths are becoming rare events [10, 16, 17]. Furthermore, it may be too late for intervention if at-risk women are identified late in labor [18]. Studies on SMM determinants will add valuable information to identify opportunities for prevention and improvements of the quality of obstetric complications at an earlier stage [19]. The purpose of this study was to determine the prevalence of SMM and associated factors. The WHO-based SMM criteria [11] were used in this study.

## Materials and methods

A hospital-based cross-sectional study was conducted at Koshi Hospital, Nepal, from January to March 2020. Morang district was chosen for its dense population, high patient flow, diversified ethnic composition, and mixed population of urban and rural areas. This facility was selected purposefully because Koshi Hospital alone covers more than 90% of the Morang district’s total deliveries (i.e., about 9000 deliveries per year) [20]. Koshi Hospital is located in an urban area in the Morang district and is a referral hospital.

The study population comprised women who gave birth in the Morang district, and the source population included all women who gave birth at Koshi Hospital. The eligible participants were women aged ≥18 years, Nepalese citizens residing in Morang district who had received routine antenatal care and had given birth at Koshi Hospital. Women more than 42 days postpartum were excluded from the study. Consecutive sampling was applied to recruit eligible participants based on the birth records at Koshi Hospital.

The sample size was calculated using Power and Sample Size Calculation software version 3.1.6 based on comparing two proportions. The proportion of SMM women without previous cesarean section experience was 13.4% [21], the proportion of SMM women with SMM was taken 28% based on expert opinion. The difference between women with and without SMM with previous cesarean section was estimated at 14.6%. The ratio of non-SMM to SMM was taken as 2:1. 95% confidence interval and 80% statistical power were used. Based on this information, the calculated sample size was 288, 96 respondents of women with SMM and 192 respondents without SMM. After considering a 20% non-response rate, the required sample size was 346.

The case report form (S1 and S2 Files) included sociodemographic information, previous obstetric history, and current obstetric conditions. Categorizations of sociodemographic variables were as follows. Ethnicity was categorized into Brahmin/Chhetri (the advantaged groups), Janajati (indigenous community), Dalits (regarded as untouchables), Muslim, and others (Marwadi), and Terai/Madhesi (native inhabitants of the flat southern region of Nepal) [22]. Religion was categorized into Hindu and Islam/others (Jain) [23]. Wealth quintiles divide the population into five quintiles (lowest, second, middle, fourth, and highest) based on the ownership of assets. The lowest quintile is the poorest population, and the highest quintile is the wealthiest population. In this study, five wealth quintiles were recategorized into lowest/second, middle, highest/fourth [23, 24]. Place of residence is an administrative division based on the population density, previous five years’ annual income, and other facilities available in the area [25]. In this study, the place of residence was categorized into the rural municipality and urban municipality. Education was categorized into no formal education/primary (1 to 5 grade), secondary (6 to 10 grade), and tertiary (11 grade and above). Occupation for women was categorized into housewife/agriculture and others (professional/managerial/self-employed) [23]. Occupation for a husband was categorized into professional/managerial/clerical, sales and services, unskilled manual/agriculture, and others [23]. Hospital records were reviewed, and face-to-face interviews were conducted. The SMM criterion was considered fulfilled if it was stated in the medical record. After childbirth, the medical records of women were retrieved retrospectively on the discharge day, to collect information on SMM conditions based on the standard WHO criteria.

The women were recruited consecutively until the required sample size was achieved. The participants were recruited daily at the postpartum ward and cabins when it was confirmed that the women had been discharged. The data were collected by a trained research assistant with an undergraduate nursing certificate supervised by the Principal Investigator (PI), a Nepalese Ph.D. candidate. After ensuring participants’ eligibility, the women were approached to enroll in the study and asked for their written informed consent. The participants’ sociodemographic characteristics and previous pregnancy history were collected via face-to-face interviews with women in a stable condition on discharge day. The criteria for the WHO SMM were checked from the medical discharge note. SMM was confirmed when a woman had at least one marker among postpartum hemorrhage, severe preeclampsia, eclampsia, sepsis or severe systemic infection, uterine rupture, or when one of the following interventions was performed: the use of blood products, laparotomy, or admission to the intensive care unit. The PI reconfirmed the data on SMM criteria.

The data were cleaned and analyzed using IBM SPSS Statistics version 26.0. The outcome variable was SMM status. The independent variables were sociodemographic variables, previous obstetric history, and current obstetric conditions. A descriptive analysis was used to determine the prevalence of SMM. The numerical variables were presented as means with standard deviations or medians with interquartile ranges. The categorical variables were presented as frequencies and percentages. A simple logistic regression analysis was performed, and all the clinically important variables or variables with p-values ≤0.30 were included in the multiple logistic exploratory regression analysis. Backward and forward methods were employed. Significant variables were analyzed for multicollinearity and interaction, and the Hosmer–Lemeshow goodness of fit test was used. The OR and 95% CI were calculated, and a p-value <0.05 was considered statistically significant.

Ethical approval was obtained from the Human Research Ethics Committee Universiti Sains Malaysia (USM/JEPeM/19060356) and the Nepal Health Research Council (Reg. no. 336/2019). The written consent of the women who agreed to participate in the study was taken before their enrolment. Permission was obtained from the hospital management to review the participants’ medical records.

## Results

A total of 346 women were included in the present study. The prevalence of SMM was 6.6%. The most frequently occurring SMM conditions were hypertensive disorders (56.5%) and hemorrhagic disorders (26.1%). Eight (34.8%) women were identified as fulfilling the severe management indicators. One early neonatal death was recorded in this study. The morbidity conditions among the women overlapped, and in total, there were 23 women with SMM (Table 1).

**Table 1:** Morbidity conditions of the women with severe maternal morbidity (n = 23).

Characteristics	n (%)
Maternal hemorrhagic disorders	*6 (26*.*1)*
Postpartum hemorrhage	6 (100.0)
Maternal hypertensive disorders	*13 (56*.*5)*
Severe hypertension	11 (84.6)
Eclampsia	2 (15.4)
Maternal severe management indicators	*8 (34*.*8)*
Prolonged hospital stays (> 7 postpartum days)	6 (75.0)
Blood transfusion	2 (25.0)

The majority of study participants were housewives (91.0%) from the Hindu religion (90.2%) with secondary or tertiary level education (72.5%). Women in this study belonged to the highest or second-highest wealth quintile (59.8%), and the majority were from Terai/Madhesi ethnicity (48.9%). Participant’s husbands also had secondary or tertiary level education (79.5%), their main occupations were unskilled manual/agriculture (42.2%) followed by sales and services (37.3%), and the majority of them were non-smokers (90.2%). The details of sociodemographic and economic characteristics and previous and current obstetric conditions of the women with and without SMM are shown in Table 2. The proportion of births by cesarean section was higher among the SMM than non-SMM women (43.5% vs. 25.7%). Nearly half (47%) of the information for the variable hemoglobin level was missing, so this variable was removed in the subsequent analysis. However, it was worth to be noted that 32% of the participants had mild (24.3%) and moderate (3.8%) anemia.

**Table 2:** Sociodemographic characteristics and previous and current obstetric conditions of the participants with and without severe maternal morbidity (n = 346).

Variables	SMM^a^ (n = 23)		non-SMM^a^ (n = 323)	
	Mean (SD)^b^	n (%)	Mean (SD)^b^	n (%)
***Sociodemographic***				
Mother’s age (year)^c^	22 (20, 24)		22 (20, 25)	
Age of marriage (year)	19.8 (2.23)		19.4 (2.38)	
Duration of marriage (year)^c^	1 (1, 4)		3 (1, 5)	
Ethnicity				
Brahmin/Chhetri		5 (21.7)		34 (10.5)
Janajati		2 (8.7)		49 (15.2)
Dalits		2 (8.7)		51 (15.8)
Muslim		1 (4.3)		33 (10.2)
Terai/Madhesi		13 (56.5)		156 (48.3)
Religion				
Hindu		22 (95.7)		290 (89.8)
Islam		1 (4.3)		33 (10.2)
Wealth quintile				
Lowest/second		1 (4.4)		19 (5.8)
Middle		9 (39.1)		110 (34.1)
Highest/fourth		13 (56.5)		194 (60.1)
Place of residence				
Rural Municipality		9 (39.1)		129 (39.9)
Urban Municipality		14 (60.9)		194 (60.1)
Mother’s education				
No formal education/primary		1 (4.3)		94 (29.1)
Secondary		17 (73.9)		159 (49.2)
Tertiary		5 (21.7)		70 (21.7)
Father’s education				
No formal education/primary		1 (4.3)		70 (21.7)
Secondary		14 (60.9)		173 (53.6)
Tertiary		8 (34.8)		80 (24.8)
Mother’s occupation				
Housewife/agriculture		21 (91.3)		294 (91.0)
Professional/managerial/self employed		2 (8.7)		29 (9.0)
Father’s occupation				
Professional/managerial/clerical		2 (8.7)		33 (10.2)
Sales and services		12 (52.2)		117 (36.2)
Unskilled manual/agriculture		8 (34.8)		138 (42.8)
Others		1 (4.3)		35 (10.8)
Father smoking habit				
No		19 (82.6)		293 (90.7)
Yes		4 (17.4)		30 (9.3)
***Previous obstetric history***				
Birth spacing (month)^c^	60 (29.7, 120)		36 (24, 60)	
Parity				
Nullipara		17 (73.9)		178 (55.1)
Multipara		6 (26.1)		145 (44.9)
Previous mode of birth				
Nulliparous		17 (73.9)		178 (55.1)
Normal birth		4 (17.4)		123 (38.1)
Cesarean section		2 (8.7)		22 (6.8)
History of abortion				
No		23 (100)		310 (96.0)
Yes		0 (0.0)		13 (4.0)
***Current obstetric conditions*** Period of gestational (week)	39.0 (1.84)		39.0 (1.73)	
Number of ANC^d^ visits				
4 visits		7 (30.4)		118 (36.5)
≤3 visits		6 (26.1)		119 (36.9)
≥5 visits		10 (43.5)		86 (26.6)
Pre-pregnancy BMI^e^ (kg/m^2^)				
Normal		19 (82.6)		244 (75.5)
Underweight		2 (8.7)		55 (17.1)
Overweight and obese		2 (8.7)		24 (7.4)
Mode of birth				
Normal birth		13 (56.5)		240 (74.3)
Cesarean section		10 (43.5)		83 (25.7)

Note:

^a^ severe maternal morbidity.

^b^ standard deviation.

^c^ median, interquartile range, skewed towards the right.

^d^ antenatal care.

^e^ body mass index.

There were 20 independent variables in this study. All the variables were analyzed using simple logistic regression to identify the factors associated with SMM (Table 3). Age of marriage, duration of marriage, mother’s education, father’s education, parity, previous mode of birth, mode of birth, and the number of ANC visits were the independent variables with *p*-value <0.3 that were analyzed in multivariate regression analysis. All independent variables that had shown significant association with SMM were tested for their collinear relationship. None of these variables were found correlated.

**Table 3:** Factors associated with severe maternal morbidity using simple logistic regression analysis (n = 346).

Variables	Crude OR^a^ (95% CI^b^)	Wald stat^c^ (df)^d^	*p*-value
***Sociodemographic***			
Mother’s age (year)	0.96 (0.85, 1.09)	0.33 (1)	0.565
Age of marriage (year)	1.07 (0.94, 1.30)	1.58 (1)	0.209
Duration of marriage (year)	0.92 (0.79, 1.07)	1.08 (1)	0.299
Ethnicity			
Brahmin/Chhetri	1.76 (0.59, 5.28)	1.03 (1)	0.310
Janajati	0.49 0.10, 2.25)	0.84 (1)	0.358
Dalits	0.47 0.10, 2.15)	0.94 (1)	0.332
Muslim	0.36 0.04, 2.88)	0.92 (1)	0.338
Terai/Madhesi	1		
Religion			
Hindu	1		
Islam	0.39 0.05, 3.06)	0.78 (1)	0.377
Wealth quintile			
Lowest/second	0.78 0.09, 6.33)	0.05 (1)	0.821
Middle	1.22 0.50, 2.94)	0.19 (1)	0.657
Highest/fourth	1		
Place of residence			
Rural Municipality	1.03 0.43, 2.46)	0.00 (1)	0.939
Urban Municipality	1		
Mother’s education			
No formal education/primary	0.09 0.01, 0.76)	4.95 (1)	0.026
Secondary	1		
Tertiary	0.69 0.24, 1.88)	0.58 (1)	0.445
Father’s education			
No formal education/primary	0.18 0.02, 1.37)	2.75 (1)	0.097
Secondary	1		
Tertiary	1.24 0.49, 3.06)	0.21(1)	0.648
Mother’s occupation			
Housewife/agriculture	1		
Professional/managerial/self-employed	0.97 0.21, 4.32)	0.00 (1)	0.963
Father’s occupation			
Professional/managerial/clerical	1.04 0.21, 5.15)	0.00 (1)	0.956
Unskilled manual/agriculture	1		
Sales and services	1.77 0.70, 4.47)	1.45 (1)	0.228
Others	0.49 0.06, 4.07)	0.43 (1)	0.511
Father smoking habit			
No	0.48 0.15, 1.52)	1.53 (1)	0.216
Yes	1		
***Previous obstetric history***			
Birth spacing (month)	1.01 0.99, 1.03)	2.59 (1)	0.107
Parity			
Nullipara	1		
Multipara	0.43 0.17, 1.13)	2.94 (1)	0.086
Previous mode of birth			
Normal birth	1		
Cesarean section	2.79 0.48, 16.20)	1.31 (1)	0.251
Nulliparous	2.93 0.96, 8.94)	3.59 (1)	0.058
History of abortion			
No	1		
Yes	0.00 0.00, 0.00)	0.00 (1)	0.999
***Current obstetric conditions*** Gestational weeks at birth (week)	1.01 0.79, 1.29)	0.00 (1)	0.923
Number of ANC^e^ visits			
4 visits	1		
≤3 visits	0.85 0.28, 2.60)	0.08 (1)	0.776
≥5 visits	1.96 0.72, 5.35)	1.72 (1)	0.189
Pre-pregnancy BMI^f^ (kg/m^2^)			
Normal	1		
Underweight	0.47 0.10, 2.06)	1.01 (1)	0.346
Overweight and obese	1.07 0.23, 4.87)	0.01 (1)	0.930
Mode of birth			
Normal birth	1		
Cesarean section	2.22 0.94, 5.26)	3.31 (1)	0.069

Note:

^a^ odds ratio.

^b^ confidence interval.

^c^ Wald statistics.

^d^ degree of freedom.

^e^ antenatal care.

^f^ body mass index.

The independent variables associated with SMM (p < 0.05) determined by multiple logistic exploratory regression analysis are shown in Table 4. The determinant found to be significantly associated with SMM was no formal and primary education (adjusted OR: 0.10, 95% CI: 0.01, 0.76). Women having no formal and primary education decreased the odds of SMM by 9 times than women with secondary education (Table 4). However, among the women with higher education, there was no significant difference in SMM status compared to women with secondary education.

**Table 4:** Factors associated with severe maternal morbidity using multiple logistic regression analysis (n = 346).

Variables	Adj. OR^a^ (95% CI^b^)	Wald stat^c^ (df^d^)	*p*-value
Maternal education			
No formal education/primary	0.10 (0.01, 0.76)	4.94 (1)	0.026
Secondary	1		
Tertiary	0.62 (0.21, 1.76)	0.80 (1)	0.370

Note:

^a^ adjusted odds ratio.

^b^ confidence interval.

^c^ Wald statistics.

^d^ degree of freedom.

## Discussions

The overall SMM ratio was 66.5/1000 deliveries in the current study. Maternal no or primary education was significantly associated with SMM. The extent of SMM has been reported in limited studies, and they are generally reported together with MNM. A wide range of SMM rates have been reported: the lowest at 1.15% in China in 2013 [26] and the highest at 19.1% in Brazil in 2017 [27]. Studies conducted among high-risk pregnant women and women with type 1 diabetes mellitus in Brazil in 2019 reported the highest rates of SMM at 35.1% [28] and 37.3% [29], respectively. The reported MNM ratios in Nepal were 22.3/1000 deliveries in 2010 [13], 3.8/1000 live births in 2013 [12], and 32.5/1000 deliveries in 2016 [30]. According to the WHO definition, SMM is lower in severity than MNM, so the higher prevalence of SMM in this study is justifiable.

The highest estimates of MNM at 198/1000 live births and 120/1000 live births have been reported in sub-Saharan Africa and the Asia region, respectively [11]. The lowest estimates reported from the Asian region were 4.4/1000 pregnancies [31] and 2.2/1000 live births [32]. The weighted pooled prevalence of MNM worldwide estimated by a systematic review and meta-analysis in 2019 was 18.67/1000 live births [33]. The vast disparities in the prevalence of MNM between high- and low-income nations could be attributed to differences in healthcare systems, particularly maternity care, study populations, and diagnostic criteria and techniques [8]. These disparities can also be explained by an investigation of a single hospital, city, or province, all of which can yield different results within the same country [11].

Maternal hemorrhagic disorders [34–36] and maternal hypertensive disorders [21, 36, 37] are the leading causes of SMM in most studies. Similarly, hypertensive disorders and hemorrhagic disorders were associated with SMM in this study. Interestingly, the literature has repeatedly noticed that hypertensive disorders are more likely to be the leading cause of SMM [10, 35, 37]. In contrast, hemorrhagic disorders are the leading cause of MNM [10, 35, 37]. It suggests that hemorrhage can occur without warning, and delays in managing hemorrhage may lead to maternal death if appropriate obstetric care is not offered, unlike hypertension that is preventable. Lotufo et al. found that hypertension was the leading cause of hospital admissions and potentially life-threatening conditions in their study; however, hemorrhage was the main cause of MNM or even death [35]. Studies have also shown that the case fatality rate of obstetric hemorrhage is higher than that of hypertensive disorders [35, 38]. Hypertensive disorders were found to cause near-miss events but not maternal deaths in a study in Pakistan; however, hemorrhage was responsible for the higher frequency of near-miss events and maternal deaths [39]. In contrast, studies have found case fatality rates higher among women with hypertensive disorders followed by severe postpartum hemorrhage [40, 41]. The higher percentages of both hypertensive disorders and hemorrhagic disorders among women indicate some form of delay in managing obstetric complications by healthcare providers [42].

In the current study, women with secondary or higher education were at 8.9 times at higher odds of developing SMM than women with no or primary education. Similar to this study, maternal higher education was significantly associated with MNM in a case-control study in southeast Iran [43]. The WHO Global Survey on Maternal and Perinatal Health, which was conducted in 24 countries, found that, compared to women with more than 12 years of education, women with no education were at a 2.7-times higher risk and women with 1–6 years of education had twice the risk of maternal mortality [44]. In the secondary data analysis of a demographic health survey in Brazil, women with no or fundamental education had 2.18 times the odds of MNM [45]. The current study findings were not in agreement with most of those in the literature.

Previous studies have shown that utilization of maternal health services increases with higher maternal education [46]. They are more likely to come from a higher socioeconomic situation, have an increased degree of health concern, and have better healthcare access [47, 48]. Women with a higher level of education are more proactive in their maternal health-seeking behavior, such as arranging prenatal checks, seeking professional health care, and likely to choose to give birth in a setting with competent medical staff and well-equipped facilities [48, 49]. In line with this evidence, in the current study, women with higher education who regularly received ANC care from Koshi Hospital identified high-risk factors. They gave birth in the same hospital. Furthermore, a slightly higher percentage of women with high blood pressure (5.2% vs. 1.1%) had a secondary or higher education than lower educated women.

On the contrary, women with no formal education lack access to health information and have no or inadequate ANC visits, which influences their awareness of obstetric complications and access to better medical services [50, 51]. The percentage of homebirths in the Morang district was 38% in the year 2016 [23]. Additionally, women with no or up to primary education were also more likely to have homebirths and have incomplete ANC. Furthermore, women with a low socioeconomic position and lack of education are more likely to wait until an emergency to seek medical help [52]. Unfortunately, these women were left out of the study, which could be one of the reasons why women with less education have a lower risk of having SMM. Approximately 44% of the women with SMM in this study had undergone a cesarean section.

In contrast, several other studies indicated higher percentages, ranging from 58.4% to 85.8% [21, 26, 35], of women who had given birth via cesarean section among MNM cases. These cesarean sections had been conducted as a required urgent action to prevent complications [37]. Although cesarean section increased the odds of SMM, an association could not be established in this study. Notwithstanding, previous studies have found cesarean section significantly associated with a higher risk of SMM [21, 37].

Studies exploring SMM determinants have found that both nulliparous and multiparous women have the highest risk of SMM [53, 54]. However, parity alone has not been shown to have a consistent association with poor obstetric outcomes [55, 56]. Nulliparous mothers are at an increased risk of hypertension [57] and, if of adolescent age, are not physically capable of childbearing [36], may delay seeking early ANC or birthing services in the event of complications [58, 59], and are in a vulnerable position concerning making decisions for themselves. Grand multipara has been reported as an independent risk factor of gestational diabetes mellitus, antepartum hemorrhage, malpresentation, and postpartum hemorrhage [55, 60].

Studies have shown that parity may be confounded by maternal age [55, 56]. Women of advanced maternal age are likely to have comorbidities [58, 61], which leaves them with less physiological reserve to cope with pregnancy morbidities [62]. Being a mother of advanced age with chronic diseases may influence the gestational prognosis, increasing the chance of complications [10]. Accordingly, parity confounded with younger or advanced age increases the likelihood of SMM. However, similar to this study, studies did not find any significant associations between SMM and maternal age or parity [8, 63]. Although nulliparous women represented 56% of the women in this study, the proportions of women ≤19 and ≥35 years were small, limiting the possibility of drawing further conclusions.

Our study findings have paved the path for referral institutions to begin routine surveillance of SMM cases using WHO criteria derived from standard medical records. Because the standard WHO criteria were followed, our findings are comparable across countries. The current study had limitations that need to be considered, i.e., it was a single hospital-based study; therefore, the findings can be generalized only to the local context in similar demographic and hospital settings. Furthermore, because homebirths, which accounted for 38 percent of births in the Morang district, were not included in the study design, the actual prevalence of SMM is predicted to be greater.

## Conclusions and recommendations

The prevalence of SMM in the current study was in line with that of other studies worldwide. Maternal lower education was associated with SMM. Women who receive routine antenatal checkups should be carefully monitored to, at a minimum, prevent hypertensive and preventable hemorrhage disorders, although postpartum hemorrhage is unpredictable. The study of SMM and its determinants can contribute to formulating strategies to prevent progression to near-miss cases and reduce maternal mortality. The study of potentially life-threatening maternal conditions can reduce future reproductive complications if health institutions know its estimate and are prepared in advance.

## Supporting information

S1 File Case report form in English. (PDF)

S2 File Case report form in Nepali. (PDF)

S3 File Severe maternal morbidity data set. (DTA)

## References

[1] World Health Organization. Trends in maternal mortality: 1990 to 2013: estimates by WHO, UNICEF, UNFPA, The World Bank and the United Nations Population Division: executive summary. World Health Organization; 2014.

[2] Camargo RS, Santana DS, Cecatti JG, Pacagnella RC, Tedesco RP, Melo EF Jr., et al. Severe maternal morbidity and factors associated with the occurrence of abortion in Brazil. Obstet Gynecol Int J. 2011;112(2):88–92. doi: 10.1016/j.ijgo.2010.08.013 21130447

[3] World Health Organization. Maternal mortality: Key facts 2020 updated 19 September 2019. https://www.who.int/news-room/fact-sheets/detail/maternal-mortality. Access Date 6/9/2020.

[4] Souza JP, Parpinelli MA, Amaral E, Cecatti JG. Population surveys using validated questionnaires provided useful information on the prevalence of maternal morbidities. J Clin Epidemiol. 2008;61(2):169–76. doi: 10.1016/j.jclinepi.2007.04.009 18177790

[5] Say L, Souza JP, Pattinson RC. Maternal near miss–towards a standard tool for monitoring quality of maternal health care. Best Pract Res Clin Obstet Gynaecol. 2009;23(3):287–96. doi: 10.1016/j.bpobgyn.2009.01.007 19303368

[6] Souza JP, Cecatti JG, Haddad SM, Parpinelli MA, Costa ML, Katz L, et al. The WHO maternal near-miss approach and the maternal severity index model (MSI): tools for assessing the management of severe maternal morbidity. PLoS One. 2012;7(8):e44129. doi: 10.1371/journal.pone.0044129 22952897 PMC3430678

[7] Giordano JC, Parpinelli MA, Cecatti JG, Haddad SM, Costa ML, Surita FG, et al. The burden of eclampsia: results from a multicenter study on surveillance of severe maternal morbidity in Brazil. PLoS One. 2014;9(5):e97401. doi: 10.1371/journal.pone.0097401 24825164 PMC4019598

[8] Pacheco AJC, Katz L, Souza ASR, de Amorim MMR. Factors associated with severe maternal morbidity and near miss in the São Francisco Valley, Brazil: a retrospective, cohort study. BMC Pregnancy Childbirth. 2014;14:91. doi: 10.1186/1471-2393-14-91 24576223 PMC3943583

[9] Geller SE, Rosenberg D, Cox SM, Brown ML, Simonson L, Driscoll CA, et al. The continuum of maternal morbidity and mortality: Factors associated with severity. Am J Obstet Gynecol. 2004;191(3):939–44. doi: 10.1016/j.ajog.2004.05.099 15467568

[10] Galvão LPL, Alvim-Pereira F, Mendonça CMM, Menezes FEF, Nascimento Góis KA, Ribeiro RF Jr, et al. The prevalence of severe maternal morbidity and near miss and associated factors in Sergipe, Northeast Brazil. BMC Pregnancy Childbirth. 2014;14(1):25. doi: 10.1186/1471-2393-14-25 24433516 PMC3898239

[11] Geller SE, Koch AR, Garland CE, MacDonald EJ, Storey F, Lawton B. A global view of severe maternal morbidity: moving beyond maternal mortality. Reprod Health. 2018;15(Suppl 1):98. doi: 10.1186/s12978-018-0527-2 29945657 PMC6019990

[12] Rana A, Baral G, Dangal G. Maternal near-miss: a multicenter surveillance in Kathmandu Valley. J Nepal Med Assoc. 2013;52(190). 24362650

[13] Shrestha N, Saha R, Karki C. Near miss maternal morbidity and maternal mortality at Kathmandu Medical College Teaching Hospital. Kathmandu Univ Med J. 2010;8(2):222–6. doi: 10.3126/kumj.v8i2.3563 21209540

[14] England N, Madill J, Metcalfe A, Magee L, Cooper S, Salmon C, et al. Monitoring maternal near miss/severe maternal morbidity: A systematic review of global practices. PLoS One. 2020;15(5):e0233697. doi: 10.1371/journal.pone.0233697 32470099 PMC7259583

[15] Tura AK, Trang TL, Van Den Akker T, Van Roosmalen J, Scherjon S, Zwart J, et al. Applicability of the WHO maternal near miss tool in sub-Saharan Africa: a systematic review. BMC Pregnancy Childbirth. 2019;19(1):79. doi: 10.1186/s12884-019-2225-7 30808325 PMC6390325

[16] Nam JY, Park E-C. The relationship between severe maternal morbidity and a risk of postpartum readmission among Korean women: a nationwide population-based cohort study. BMC Pregnancy Childbirth. 2020;20(1):1–8. doi: 10.1186/s12884-020-2820-7 32143586 PMC7060630

[17] Callaghan WM, Grobman WA, Kilpatrick SJ, Main EK, D’Alton M. Facility-based identification of women with severe maternal morbidity: it is time to start. Obstet Gynecol. 2014;123(5):978. doi: 10.1097/AOG.0000000000000218 24785849 PMC4293012

[18] Easter SR, Bateman BT, Sweeney VH, Manganaro K, Lassey SC, Gagne JJ, et al. A comorbidity-based screening tool to predict severe maternal morbidity at the time of delivery. Am J Obstet Gynecol 2019;221(3):271. e1–. e10. 31229427 10.1016/j.ajog.2019.06.025

[19] Gibson C, Rohan AM, Gillespie KH. Severe maternal morbidity during delivery hospitalizations. WMJ. 2017;116(5):215. 29357211 PMC6366334

[20] Morang District Health Office. Annual Health Report, 2074/075 (2017/2018). Province Government, Province No 1, Ministry of Social Development2019.

[21] Norhayati MN, Nik Hazlina NH, Aniza AA, Sulaiman Z. Factors associated with severe maternal morbidity in Kelantan, Malaysia: A comparative cross-sectional study. BMC Pregnancy Childbirth. 2016;16(1):185. doi: 10.1186/s12884-016-0980-2 27460106 PMC4962372

[22] Bennett L, Dahal DR, Govindasamy P. Caste, Ethnic and Regional Identity in Nepal: Further Analysis of the 2006 Nepal Demographic and Health Survey. Calverton, Maryland,: USA: Macro International Inc.; 2008.

[23] Ministry of Health Nepal, New ERA, ICF. Nepal Demographic and Health Survey 2016. Kathmandu, Nepal: Ministry of Health, Nepal; 2017.

[24] Fry K, Firestone R, Chakraborty NM. Measuring Equity with Nationally Representative Wealth Quintiles Washington, DC: PSI; 2014. https://www.psi.org/wp-content/uploads/2014/10/Wealth-Quintile-Guide.pdf. Access Date 6 May.

[25] Ministry of General Affairs and General Administration. Local Government Operation Act, 2074 Kathmandu, Nepal 2017. https://mofaga.gov.np/news-notice/1697. Access Date 29 April, 2021.

[26] Shen FR, Liu M, Zhang X, Yang W, Chen YG. Factors associated with maternal near‐miss morbidity and mortality in Kowloon Hospital, Suzhou, China. Obstet Gynecol Int J. 2013;123(1):64–7. 10.1016/j.ijgo.2013.06.011 23886451

[27] Lima HM, Carvalho FHC, Feitosa FEL, Nunes GC. Factors associated with maternal mortality among patients meeting criteria of severe maternal morbidity and near miss. Obstet Gynecol Int J. 2017;136(3):337–43. doi: 10.1002/ijgo.12077 28099693

[28] de Lima THB, Amorim MM, Kassar SB, Katz L. Maternal near miss determinants at a maternity hospital for high-risk pregnancy in northeastern Brazil: a prospective study. BMC Pregnancy Childbirth. 2019;19(1):271. doi: 10.1186/s12884-019-2381-9 31370813 PMC6670122

[29] Morais LR, Patz B, Campanharo FF, Sun S. Maternal near miss and Potentially Life-Threatening Condition Determinants in Patients with type 1 Diabetes Mellitus in a University Hospital in Sao Paulo, Brazil: A Retrospective Study. 2020. 10.1186/s12884-020-03392-y PMC7653718 33172430

[30] Bajagain R, Saha RS. Incidence and predictors of severe obstetric morbidity in a teaching hospital. JKMC. 2016;5(2):44–8.

[31] Soma-Pillay P, Pattinson RC, Langa-Mlambo L, Nkosi BS, MacDonald AP. Maternal near miss and maternal death in the Pretoria Academic Complex, South Africa: A population-based study. S Afr Med J. 2015;105(7):578–83. doi: 10.7196/SAMJnew.8038 26428756

[32] Norhayati MN, Hazlina NHN, Sulaiman Z, Azman MY. Severe maternal morbidity and near misses in tertiary hospitals, Kelantan, Malaysia: a cross-sectional study. BMC Public Health. 2016;16(1):229. doi: 10.1186/s12889-016-2895-2 26944047 PMC4779219

[33] Abdollahpour S, Miri HH, Khadivzadeh T. The global prevalence of maternal near miss: a systematic review and meta-analysis. Health Promot Perspect. 2019;9(4):255. doi: 10.15171/hpp.2019.35 31777704 PMC6875559

[34] Grobman WA, Bailit JL, Rice MM, Wapner RJ, Reddy UM, Varner MW, et al. Frequency of and factors associated with severe maternal morbidity. Obstet Gynaecol. 2014;123(4):804. doi: 10.1097/AOG.0000000000000173 24785608 PMC4116103

[35] Lotufo FA, Parpinelli MA, Haddad SM, Surita FG, Cecatti JG. Applying the new concept of maternal near-miss in an intensive care unit. Clinics. 2012;67(3):225–30. doi: 10.6061/clinics/2012(03)04 22473402 PMC3297030

[36] Mekango DE, Alemayehu M, Gebregergs GB, Medhanyie AA, Goba G. Determinants of maternal near miss among women in public hospital maternity wards in Northern Ethiopia: A facility based case-control study. PLoS One. 2017;12(9):e0183886. doi: 10.1371/journal.pone.0183886 28886034 PMC5590854

[37] Madeiro AP, Rufino AC, Lacerda ÉZG, Brasil LG. Incidence and determinants of severe maternal morbidity: a transversal study in a referral hospital in Teresina, Piaui, Brazil. BMC Pregnancy Childbirth. 2015;15(1):210. 26347370 10.1186/s12884-015-0648-3 PMC4562200

[38] Nakimuli A, Nakubulwa S, Kakaire O, Osinde MO, Mbalinda SN, Kakande N, et al. The burden of maternal morbidity and mortality attributable to hypertensive disorders in pregnancy: a prospective cohort study from Uganda. BMC Pregnancy Childbirth. 2016;16(1):205. doi: 10.1186/s12884-016-1001-1 27492552 PMC4973370

[39] Mustafa R, Hashmi H. Near-miss obstetrical events and maternal deaths. J Coll Physicians Surg Pak. 2009;19(12):781–5. 20042157

[40] Moodley J. Maternal deaths associated with hypertension in South Africa: lessons to learn from the Saving Mothers report, 2005–2007. Cardiovasc J Afr. 2011;22(1):31. doi: 10.5830/cvja-2010-042 21298203 PMC3734738

[41] Sayinzoga F, Bijlmakers L, van der Velden K, van Dillen J. Severe maternal outcomes and quality of care at district hospitals in Rwanda–a multicentre prospective case-control study. BMC Pregnancy Childbirth. 2017;17(1):394. doi: 10.1186/s12884-017-1581-4 29178885 PMC5702108

[42] Liyew EF, Yalew AW, Afework MF, Essén B. Incidence and causes of maternal near-miss in selected hospitals of Addis Ababa, Ethiopia. PLoS One. 2017;12(6):e0179013. doi: 10.1371/journal.pone.0179013 28586355 PMC5460898

[43] Naderi T, Foroodnia S, Omidi S, Samadani F, Nakhaee N. Incidence and correlates of maternal near miss in Southeast Iran. Int J Reprod Med. 2015;2015. doi: 10.1155/2015/914713 25763409 PMC4345045

[44] Karlsen S, Say L, Souza J-P, Hogue CJ, Calles DL, Gülmezoglu AM, et al. The relationship between maternal education and mortality among women giving birth in health care institutions: analysis of the cross sectional WHO Global Survey on Maternal and Perinatal Health. BMC Public Health. 2011;11(1):606. 21801399 10.1186/1471-2458-11-606 PMC3162526

[45] Souza J, Cecatti J, Parpinelli M, Sousa M, Lago T, Pacagnella R, et al. Maternal morbidity and near miss in the community: findings from the 2006 Brazilian demographic health survey. BJOG. 2010;117(13):1586–92. doi: 10.1111/j.1471-0528.2010.02746.x 21078054

[46] Wang H, Frasco E, Takesue R, Tang K. Maternal education level and maternal healthcare utilization in the Democratic Republic of the Congo: an analysis of the multiple indicator cluster survey 2017/18. BMC Health Serv Res. 2021 2021/08/21;21(1):850. doi: 10.1186/s12913-021-06854-x 34419033 PMC8380349

[47] Gabrysch S, Campbell OM. Still too far to walk: literature review of the determinants of delivery service use. BMC Pregnancy Childbirth. 2009;9(1):34. doi: 10.1186/1471-2393-9-34 19671156 PMC2744662

[48] Ahmed S, Creanga AA, Gillespie DG, Tsui AO. Economic Status, Education and Empowerment: Implications for Maternal Health Service Utilization in Developing Countries. PLoS ONE. 2010;5(6):e11190. doi: 10.1371/journal.pone.0011190 20585646 PMC2890410

[49] Papiernik E, Tafforeau J, Richard A, Pons J-C, Keith LG. Perception of risk, choice of maternity site, and socio economic level of twin mothers. J Perinat Med. 1997;25(2):139–45. doi: 10.1515/jpme.1997.25.2.139 9189833

[50] Dessalegn FN, Astawesegn FH, Hankalo NC. Factors associated with maternal near miss among women admitted in West Arsi Zone public hospitals, Ethiopia: Unmatched case-control study. J Pregnancy. 2020;2020:6029160-. doi: 10.1155/2020/6029160 32695514 PMC7352151

[51] Rajbanshi S, Norhayati MN, Nik Hazlina NH. Risk perceptions among high-risk pregnant women in Nepal: a qualitative study. BMC Pregnancy Childbirth. 2021 2021/08/04;21(1):539. doi: 10.1186/s12884-021-04018-7 34348703 PMC8340362

[52] Rajbanshi S, Norhayati MN, Nik Hazlina NH. A Qualitative Study to Explore the Barriers for Nonadherence to Referral to Hospital Births by Women with High-Risk Pregnancies in Nepal. IJERPH. 2021;18(11). doi: 10.3390/ijerph18115801 34071394 PMC8199227

[53] Nansubuga E, Ayiga N, Moyer CA. Prevalence of maternal near miss and community-based risk factors in Central Uganda. BJOG. 2016;135(2):214–20. doi: 10.1016/j.ijgo.2016.05.009 27553504

[54] Zwart J, Richters J, Öry F, De Vries J, Bloemenkamp K, Van Roosmalen J. Severe maternal morbidity during pregnancy, delivery and puerperium in the Netherlands: a nationwide population-based study of 371 000 pregnancies. BJOG. 2008;115(7):842–50. doi: 10.1111/j.1471-0528.2008.01713.x 18485162

[55] Mgaya AH, Massawe SN, Kidanto HL, Mgaya HN. Grand multiparity: is it still a risk in pregnancy? BMC Pregnancy Childbirth. 2013;13(1):241. 24365087 10.1186/1471-2393-13-241 PMC3878019

[56] Bai J, Wong FW, Bauman A, Mohsin M. Parity and pregnancy outcomes. Am J Obstet Gynecol. 2002;186(2):274–8. doi: 10.1067/mob.2002.119639 11854649

[57] Gupta S, Yamada G, Mpembeni R, Frumence G, Callaghan-Koru JA, Stevenson R, et al. Factors associated with four or more antenatal care visits and its decline among pregnant women in Tanzania between 1999 and 2010. PLoS One. 2014;9(7):e101893. doi: 10.1371/journal.pone.0101893 25036291 PMC4103803

[58] Oliveira FC, Surita FG, e Silva JLP, Cecatti JG, Parpinelli MA, Haddad SM, et al. Severe maternal morbidity and maternal near miss in the extremes of reproductive age: results from a national cross-sectional multicenter study. BMC Pregnancy Childbirth. 2014;14(1):77. doi: 10.1186/1471-2393-14-77 24555831 PMC3975952

[59] Ochako R, Fotso J-C, Ikamari L, Khasakhala A. Utilization of maternal health services among young women in Kenya: insights from the Kenya Demographic and Health Survey, 2003. BMC Pregnancy Childbirth. 2011;11(1):1. doi: 10.1186/1471-2393-11-1 21214960 PMC3022772

[60] Al J. Grandmultiparity: a potential risk factor for adverse pregnancy outcomes. J Reprod Med. 2012;57(1–2):53–7. 22324269

[61] Gray KE, Wallace ER, Nelson KR, Reed SD, Schiff MA. Population-based study of risk factors for severe maternal morbidity. Paediatr Perinat Epidemiol. 2012;26(6):506–14. doi: 10.1111/ppe.12011 23061686 PMC3498497

[62] Kayem G, Kurinczuk J, Lewis G, Golightly S, Brocklehurst P, Knight M. Risk Factors for Progression from Severe Maternal Morbidity to Death: A National Cohort Study. PLoS One. 2011;6(12):e29077. doi: 10.1371/journal.pone.0029077 22216171 PMC3247232

[63] Hitti J, Sienas L, Walker S, Benedetti TJ, Easterling T. Contribution of hypertension to severe maternal morbidity. Am J Obstet Gynecol. 2018;219(4):405. e1–. e7. doi: 10.1016/j.ajog.2018.07.002 30012335

