# Supplementary material for: Severe maternal morbidity and its associated factors: A cross-sectional study in Morang district, Nepal
Source: PLoS One. 2021 Dec 31;16(12):e0261033. doi: 10.1371/journal.pone.0261033 (PMC8719668; doi:10.1371/journal.pone.0261033)
Supplement: S1 File — (PDF) [file pone.0261033.s001.pdf]

Case Report Form 1

Date: ________________

Inclusion criteria:
Woman aged ≥18 years ☐Yes ☐No
29 to 32 weeks of pregnancy ☐Yes ☐No

Exclusion criteria:
> 42 days of termination of pregnancy ☐Yes ☐No

Section A: Respondent

<table>
<tr><td colspan="2">ID No. _______ Unique ID Code. _____</td></tr>
<tr><td>Ethnicity: ___________________________</td><td>Bed no: ________</td></tr>
<tr><td>Date of admission: ________________</td><td>Date of discharge: ________________</td></tr>
<tr><td>Duration of hospitalization: __________</td><td></td></tr>
<tr><td>Date of birth: ____________________</td><td>EDD: ________________</td></tr>
<tr><td>Gestation weeks _________</td><td></td></tr>
<tr><td colspan="2">Address: ____________________ Tole Name ____________ Ward no: ____________
☐ Nagarpalika
☐ Gaunpalika</td></tr>
<tr><td colspan="2">Phone number (mobile) : _____________________________ ☐self ☐husband</td></tr>
</table>

Section B: Sociodemographic data

SN	Questions	Options
101	Ethnicity	1. Brahmin/Chettri<br>2. Terai/Madhesi other castes<br>3. Dalits<br>4. Newar<br>5. Janajati<br>6. Muslim<br>7. Others
102	Religion	1. Hindu<br>2. Buddhist<br>3. Muslim<br>4. Kirat<br>5. Christian<br>6. Other
103	Woman's highest education achieved	………… passed grade

104	Husband highest education achieved	………… passed grade
105	Woman occupation	1. Housewife<br>2. Self-employed<br>3. Professional/managerial<br>4. Agriculture<br>5. Unskilled manual<br>6. Others …………. specify
106	Husband occupation	1. Professional technical/managerial<br>2. Clerical<br>3. Sales and services<br>4. Skilled manual<br>5. Unskilled manual<br>6. Agriculture<br>7. Others …………. specify
107	Husband smoke	1. Yes …………. sticks/day<br>2. No
108	Completed age	_____years
109	Age at marriage	_____ years
110	Years of marriage	_____ years

Section C: Equity Tool

SN	Questions	Options
201	Does your household have a television?	1. Yes<br>2. No
202	Does your household have a cupboard?	1. Yes<br>2. No
203	Does your household have a table?	1. Yes<br>2. No
204	Does your household have a fan?	1. Yes<br>2. No
205	What is the main material of the floor of your dwelling?	1. Earth/sand<br>2. Other
206	What is the main material of the exterior walls of your dwelling?	1. Cement<br>2. Other
207	What is the main material of the roof of your dwelling?	1. Cement<br>2. Other
208	Why type of fuel does your household mainly use for cooking?	1. LPG<br>2. Wood<br>3. Other

Section D: Past Obstetric history

SN	Past Obstetric history	Options
301	Number of children	_______ None _________ Boys _______ Girls
302	Birth spacing in the last pregnancy	_________ months
303	Mode of delivery for the last pregnancy	☐ Emergency caesarean section<br>☐ Elective caesarean section<br>☐ Assisted vaginal delivery<br>☐ Spontaneous vaginal delivery
304	Complications in the previous pregnancy	☐ Yes, ________________ specify<br>☐ No
305	Number of abortions	☐ Yes, ____________ times<br>☐ No

Section E: Current Obstetric conditions

SN	Current obstetric conditions	Options
401	Pre-pregnancy BMI	________ kg/m^2
402	Number of antenatal visits done before today's visit	_________ times
403	Problems detected	______________ (specify)<br>______________ (specify)<br>______________ (specify)<br>______________ (specify)
404	Color codes	☐ Red<br>☐ Yellow<br>☐ Green<br>☐ White
405	Mode of delivery	☐ Spontaneous vaginal delivery<br>☐ Assisted vaginal delivery<br>☐ Emergency caesarean section<br>☐ Elective caesarean section<br>☐ Others _____________ (specify)

Section F: Clinical parameters

SN	Clinical parameters	Options
501	Blood pressure	systolic ________ mm/Hg<br>diastolic ________ mm/Hg
502	Haemoglobin, Hb	________________ g/dL

Section G: Severe maternal morbidity

**Haemorrhagic disorders**
☐ Abruptio placentae<br>☐ Placenta accreta/increta/percreta<br>☐ Ectopic pregnancy<br>☐ Postpartum haemorrhage<br>☐ Ruptured uterus
**Hypertensive disorders**
☐ Severe pre-eclampsia<br>☐ Eclampsia<br>☐ Severe hypertension<br>☐ Hypertensive encephalopathy<br>☐ HELLP (haemolysis, elevated liver enzymes, low platelet count) syndrome
**Other systemic disorders**
☐ Endometritis<br>☐ Pulmonary oedema<br>☐ Respiratory failure<br>☐ Seizures<br>☐ Sepsis<br>☐ Shock<br>☐ Thrombocytopenia (< 100,000)<br>☐ Thyroid crisis
**Severe management indicators**
☐ Blood transfusion<br>☐ Central venous access<br>☐ Hysterectomy<br>☐ Intensive care unit admission<br>☐ Prolonged hospital stay (> 7 postpartum days)<br>☐ Intubation not related to anaesthetic procedure<br>☐ Return to operating room

☐ Laparotomy (includes hysterectomy, excludes caesarean section)
