# Supplementary material for: Severe maternal morbidity and its associated factors: A cross-sectional study in Morang district, Nepal
Source: PLoS One. 2021 Dec 31;16(12):e0261033. doi: 10.1371/journal.pone.0261033 (PMC8719668; doi:10.1371/journal.pone.0261033)
Supplement: S2 File — (PDF) [file pone.0261033.s002.pdf]

केस रिपोर्ट फारम १ ....

मितिः ....

समावेश गर्नका लागि मापदण्ड:

≥18 वर्ष उमेरका महिला ☐ हो ☐ होइन
२९ देखि ३२ हप्तासम्मको गर्भवति ☐ हो ☐ होइन

बहिष्करण मापदण्ड:
सुत्केरी भएको ४२ दिन भन्दा बढि ☐ हो ☐ होइन

खण्ड कः सहभागी

आईडी नम्बर. _______	
जात: ____________________________	बेड नं: _______________
अस्पतालमा भर्ना गरेको मिति: ________________	अस्पतालबाट डिस्चार्ज गरेको मिति: ________________
अस्पतालमा बसेको दिन: _________________	
जन्म मिति: ____________________	EDD: _________________
गर्भ रहेको महिना : _________ हप्तामा	
ठेगाना: ____________________ टोलको नाम: __________________ वार्ड नं: __________________<br>☐ नगरपालिका<br>☐ गाउपालिका	
फेन नं मोबाइल : ___________________________ ☐ आफ्नै ☐ श्रीमानको	

खण्ड ख : सामाजिक तथा जनसांख्यि

सि. नं.	प्रश्नहरू	उत्तरहरू
101	जाती	1. ब्राह्मण / क्षेत्री<br>2. तराई / मशीशी अन्य जातिहरू<br>3. दलित<br>4. नेवार<br>5. जनजाती<br>6. मुस्लिम<br>7. अन्य
102	धर्म	1. हिन्दू<br>2. बौद्ध<br>3. मुस्लिम<br>4. किटैट<br>5. ईसाई<br>6. अन्य
103	महिलाले पास गरेको शिक्षा	.... कक्षा
104	पुरूषले पास गरेको शिक्षा	.... कक्षा

105	महिलाको पेशा	1. गृहिणी<br>2. स्व-रोजगारी<br>3. व्यावसायिक / प्रबन्धकीय<br>4. कृषि<br>5. तालिम बिनाको ज्याला दारी काम<br>6. अन्य ....
106	पुरूषको पेशा	1. व्यावसायिक प्राविधिक / प्रबन्धकीय<br>2. जागीर<br>3. व्यवसाय र सेवाहरू<br>4. तालीम ज्याला दारी<br>5. तालिम बिनाको ज्याला दारी काम<br>6. कृषि<br>7. अन्य ....
107	श्रीमानले चुरोट खान्छ	1. खान्छ .... दिनमा कति वटा खिल्ली खान्छ<br>2. खान्दैन
109	पुरा भएको उमेर	.... वर्ष
110	विवाह हुदाँको उमेर	.... वर्ष
111	विवाह भएको उमेर	.... वर्ष

खण्ड ग : धनको सुचक

सि. नं.	प्रश्नहरू	उत्तरहरू
201	टि.भि	1. छ<br>2. छैन
202	डराज	1. छ<br>2. छैन
203	टेबल	1. छ<br>2. छैन
204	पखां	1. छ<br>2. छैन
205	घरको भूइ के को छ	1. Earth/sand<br>2. अन्य
206	घरको बाहिरी भित्ता के को छ	1. सिमेन्ट<br>2. अन्य
207	घरको छत के को छ	1. सिमेन्ट<br>2. अन्य
208	खाना पकाउन कुन इन्धन प्रयोग गर्छ	1. LPG<br>2. काठ<br>3. अन्य

खण्ड घ : पछिल्लो गर्भ सम्बन्धि जानकारी

सि. नं.	प्रश्नहरू	उत्तरहरू
301	बच्चाको संख्या	☐ छैन _______ केटा _______ केटी

302	पछिल्लो बच्चा र यो गर्भको बीचमा अन्तराल	_______ महिना
303	पछिल्लो प्रशुति कसरी भएको थियो ?	☐ Emergency caesarean section<br>☐ Elective caesarean section<br>☐ Assisted vaginal delivery<br>☐ Spontaneous vaginal delivery
304	पछिल्लो प्रशुतिमा केही जतिलता देखिएको थियो ?	☐ थियो, ______________ specify<br>☐ थिएन
305	यदि गर्भपतन गरेको भए, कति पटक गरेको	☐ थियो,__________ पटक<br>☐ थिएन

खण्ड ङ : हालको गर्भ अवस्था

सि. नं.	प्रश्नहरू	उत्तरहरू
401	Pre-pregnancy BMI	______ kg/m2
402	Number of antenatal visits done before today's visit	_______ times
403	Problems detected	___________ (specify)<br>___________ (specify)<br>___________ (specify)<br>___________ (specify)
404	Color code	☐ Red<br>☐ Yellow<br>☐ Green<br>☐ White
405	Mode of delivery	☐ Spontaneous vaginal delivery<br>☐ Assisted vaginal delivery<br>☐ Emergency caesarean section<br>☐ Elective caesarean section<br>☐ Others __________ (specify)

खण्ड च : क्लिनिकल प्यारामिटरहरू

सि. नं.	क्लिनिकल प्यारामिटरहरू	उत्तरहरू
501	Blood pressure	systolic _________ mm/Hg<br>diastolic _________ mm/Hg
502	Haemoglobin, Hb	__________________ g/dL

खण्ड छ : Severe maternal morbidity

**Haemorrhagic disorders**
☐ Abruptio placentae<br>☐ Placenta accreta/increta/percreta<br>☐ Ectopic pregnancy<br>☐ Postpartum haemorrhage<br>☐ Ruptured uterus
**Hypertensive disorders**
☐ Severe pre-eclampsia<br>☐ Eclampsia<br>☐ Severe hypertension<br>☐ Hypertensive encephalopathy<br>☐ HELLP (haemolysis, elevated liver enzymes, low platelet count) syndrome
**Other systemic disorders**
☐ Endometritis<br>☐ Pulmonary oedema<br>☐ Respiratory failure<br>☐ Seizures<br>☐ Sepsis<br>☐ Shock<br>☐ Thrombocytopenia (< 100,000)<br>☐ Thyroid crisis
**Severe management indicators**
☐ Blood transfusion<br>☐ Central venous access<br>☐ Hysterectomy<br>☐ Intensive care unit admission<br>☐ Prolonged hospital stay (> 7 postpartum days)<br>☐ Intubation not related to anaesthetic procedure<br>☐ Return to operating room<br>☐ Laparotomy (includes hysterectomy, excludes caesarean section)
